# The environmentally responsive plant epigenome: insights from jasmonate signaling

**DOI:** 10.1111/nph.70865

**Published:** 2025-12-21

**Authors:** Mark Zander, Emily Vesper

**Affiliations:** ^1^ Waksman Institute of Microbiology, Department of Plant Biology Rutgers, The State University of New Jersey 190 Frelinghuysen Rd Piscataway NJ 08854‐8020 USA

**Keywords:** 3D chromatin organization, chromatin landscapes, epigenomes, histone modifications, jasmonate signaling, transcription factors

## Abstract

The environmental responsiveness of the plant epigenome is essential for spatiotemporally precise gene regulation, enabling plants to adapt to external cues. Elucidating the mechanisms underlying this responsiveness is therefore fundamental to deciphering the molecular logic of plant‐environment interactions. In this review, we highlight the dynamic regulation of the plant epigenome by the hormone jasmonic acid (JA), which orchestrates immune and developmental responses. Our understanding of JA‐induced epigenome reprogramming has expanded significantly in recent years, and these insights can serve as a blueprint for other environmental response pathways. The hallmarks of an environmentally responsive epigenome will be emphasized, focusing on the roles of transcription factors as epigenome architects and on three‐dimensional chromatin reorganization as an emerging hallmark of epigenome responsiveness. We envision that the general principles of cue‐induced epigenome reprogramming outlined here will guide future studies across diverse cues and species.

**Table:** 

	Contents	
	Summary	2722
I.	Introduction	2722
II.	Hallmarks of the JA‐responsive epigenome	2723
III.	Regulators of epigenome responsiveness	2725
IV.	Conclusions	2726
V.	Perspectives	2727
	Acknowledgements	2727
	References	2727

## I. Introduction

The integration of environmental cues into gene regulatory networks (GRNs) underpins plant adaptability and survival. Central to this cue‐dependent integration is the epigenome which encompasses chemical modifications of chromatin (DNA methylation and post‐translational modifications (PTMs) of histones), chromatin accessibility, three‐dimensional (3D) chromatin organization, and long noncoding RNAs (lncRNAs). We use the responsiveness of the epigenome to the plant hormone jasmonic acid (JA) as an example to illustrate the general principles by which plants integrate environmental cues into their epigenomic landscape.

JA is synthesized in response to wounding and pathogen infection, as well as herbivory, and is frequently used directly in experimental studies. Its bioactive conjugate, (+)‐7‐iso‐jasmonoyl‐L‐isoleucine (JA‐Ile), is perceived on chromatin by the JA‐Ile co‐receptor complex, which consists of the F‐box protein CORONATINE INSENSITIVE 1 (COI1) and JASMONATE‐ZIM DOMAIN (JAZ) repressor proteins. COI1 acts as part of the Skp‐Cullin F‐box E3 ubiquitin ligase complex (SCF^COI1^), which ubiquitinates JAZ proteins upon JA‐Ile binding, targeting them for proteasomal degradation. In the absence of JA‐Ile, JAZs repress the master transcription factors (TFs): the basic helix–loop–helix TFs MYELOCYTOMATOSIS 2 (MYC2) and its closest homologs MYC3, MYC4, and MYC5 (MYCs; Qi *et al*., 2015; Lorenzo *et al*., 2004; Fernandez‐Calvo *et al*., 2011; Song *et al*., 2017). Liberation from JAZs allows MYCs to activate a large immune GRN with thousands of genes transcriptionally reprogrammed (Du *et al*., 2017; Wang *et al*., 2019; Zander *et al*., 2020; Choudhary *et al*., 2024).

Here, we shed light on the epigenome changes that occur once MYCs are liberated and start activating the JA pathway in the model species *Arabidopsis thaliana*. We first outline the defining hallmarks of the JA‐responsive epigenome and then dissect the molecular mechanisms underlying its establishment. Finally, we outline general principles governing epigenome responsiveness to JA that extend to other environmental signaling pathways.

## II. Hallmarks of the JA‐responsive epigenome

**Fig. 1:**
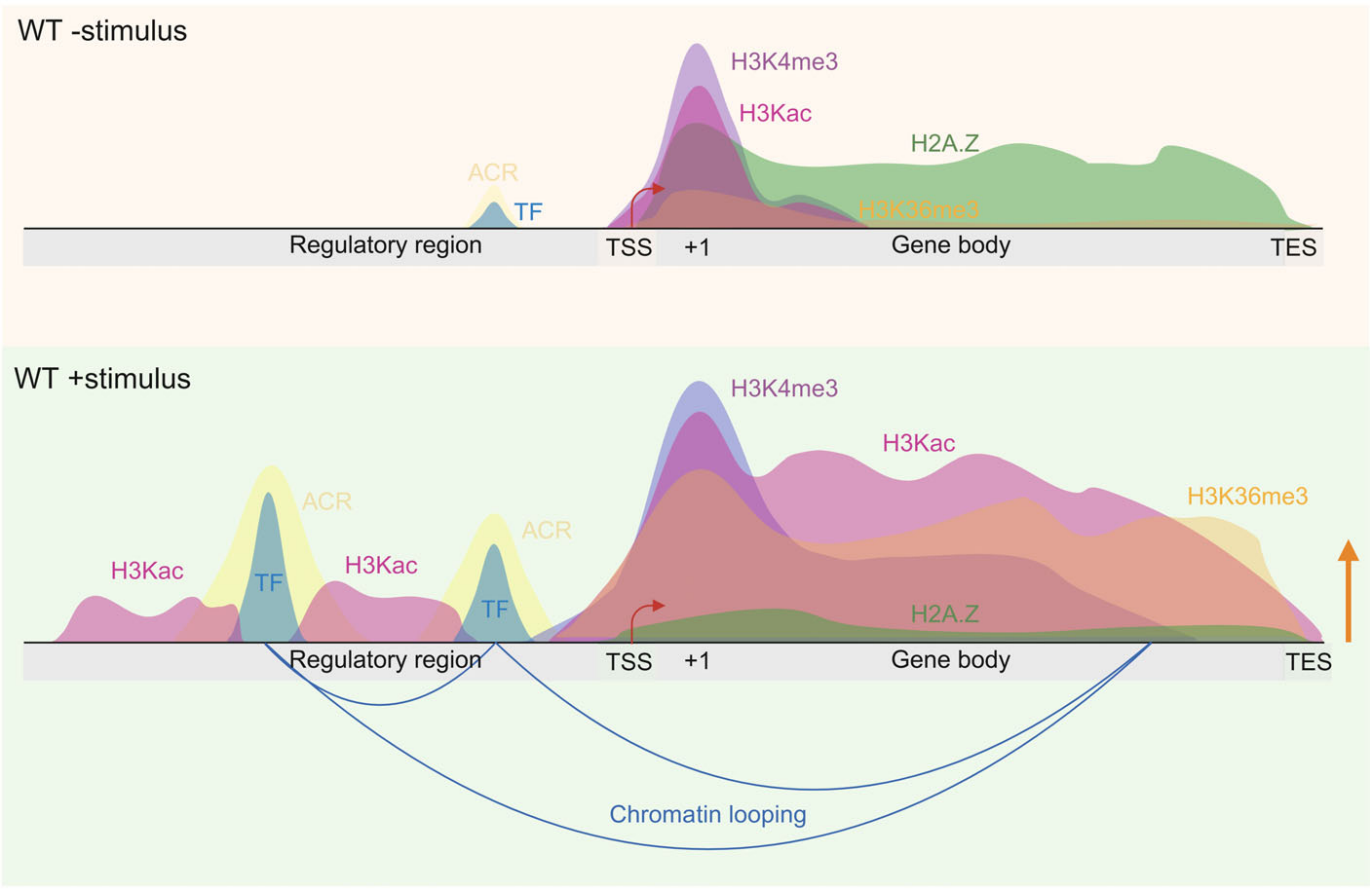
Hallmarks of an environmentally responsive gene‐level epigenome. Schematic illustration of a genome browser snapshot highlighting major epigenome changes that occur at an environmentally responsive gene following cue perception. Histone post‐translational modification (PTM) domains, transcription factor (TF) binding peaks, and accessible chromatin regions (ACRs) are modeled after representative genomic datasets (chromatin immunoprecipitation sequencing (ChIP‐seq) for histone PTMs, histone variants, and TF binding; Assay for Transposase‐Accessible Chromatin using sequencing (ATAC‐seq) for ACRs). In the absence of a cue (upper panel), only weak TF binding is observed within a small ACR in the regulatory region. The gene body exhibits modest levels of active histone marks H3K4me3, H3Kac (representative for acetylation of histone 3 lysine 9 (H3K9ac), H3K14ac, and H3K27ac), and low H3K36me3 enrichment around the +1 nucleosome indicated with +1. By contrast, the repressive histone variant H2A.Z is abundant throughout the gene body. Cue perception triggers strong TF binding at multiple *cis*‐regulatory elements (CREs), resulting in expanded ACRs. Levels of H3K4me3, H3Kac, and H3K36me3 increase both near the +1 nucleosome and across the gene body. H3Kac is also detected within Stimulus‐Induced Enhancer Acetylation (SIENA) regions surrounding TF binding sites in the regulatory region. Chromatin looping is also established, bringing CREs into proximity with the gene body. Meanwhile, levels of gene body‐localized H2A.Z decrease substantially. Collectively, these cue‐induced chromatin changes promote robust gene activation, illustrated by the orange arrow. This figure was created in BioRender (https://BioRender.com/emev1ax).

The hallmarks of cue‐induced epigenome reprogramming highlighted here for JA signaling are also applicable to other response pathways (Fig. 1; Table 1). Histone tail acetylation of histones H3 (H3ac) and H4 (H4ac) weakens the interactions between histone tails and nucleosomal DNA, thereby increasing chromatin accessibility for the transcriptional machinery (Chen *et al*., 2024). These histone PTMs were the earliest epigenome features linked to JA signaling following discoveries that the histone deacetylase (HDAC) HISTONE DEACETYLASE 6 (HDA6) can interact with COI1, that HDA6 and HDA19 regulate JA‐induced gene expression, and that NOVEL INTERACTOR OF JAZ (NINJA) links JAZs to the transcriptional corepressor TOPLESS (TPL), known to associate with various HDACs (Devoto *et al*., 2002; Zhou *et al*., 2005; Wu *et al*., 2008; Pauwels *et al*., 2010; Krogan *et al*., 2012). The first gene‐specific evidence was detected for JA‐induced H4ac at the *ETHYLENE RESPONSE FACTOR 1* (*ERF1*) gene and later JA‐induced acetylation of histone 3 lysine 9 (H3K9ac) at other JA‐inducible marker genes (Zhu *et al*., 2011; You *et al*., 2019; Li *et al*., 2020). Subsequent JA‐Ile time‐series experiments revealed that H3K9ac levels gradually increase at *ERF1* and *JAZ8* over time (An *et al*., 2017). Moreover, a H4ac chromatin immunoprecipitation sequencing (ChIP‐seq) analysis demonstrated on a genome‐wide scale that genes transcriptionally induced by JA substantially overlap with genes enriched for JA‐induced H4ac near their transcription start sites (Vincent *et al*., 2022).

**Table 1:** Overview of jasmonic acid (JA)‐responsive epigenome features.

Feature	Localization	Function	JA impact	Writer	Eraser	Species	References
H3ac	TSS, gene body	Gene activation	Increase	(+JA), HAT: HAC1 and unknown HATs	(−JA), HDAC: unknown	*Arabidopsis, tomato*	An *et al*. (2017), Choudhary *et al*. (2024)
H3K9ac	Regulatory region	Unknown	Increase	(+JA), HAT: HAC1 and unknown HATs	(−JA), HDAC: unknown	*Arabidopsis, tomato*	Choudhary *et al*. (2024)
H3K4me3	TSS, gene body	Gene activation	Increase	(+ JA), HMT: SDG33/34	(−JA), HDM: unknown	*Arabidopsis, tomato*	Bvindi *et al*. (2022)
H3K36me3	TSS, gene body	Transcription elongation	Increase	(+ JA), HMT: SDG8/33/34	(−JA), HDM: unknown	*Arabidopsis, tomato*	Berr *et al*. (2010), Zhang *et al*. (2019), Bvindi *et al*. (2022)
H3K27me3	Entire gene	Gene repression	Decrease	(+JA), HMT: PRC2 complex	(−JA), HDM: unknown	*Arabidopsis*	Ramirez‐Prado *et al*. (2019), Li *et al*. (2021)
H2A.Z	TSS, gene body	Gene repression	Decrease	(+JA), CRC: unknown	(−JA), CRC: SWR1 complex	*Arabidopsis*	Coleman‐Derr & Zilberman (2012), Berriri *et al*. (2016)
DNA methylation	TEs	TE silencing	Decrease	(+JA), DNMT: DRM2	(−JA), DNML: ROS1	*Arabidopsis, tomato*	Wilkinson *et al*. (2023), Du *et al*. (2025), Zhang *et al*. (2025)

The table summarizes epigenomic features with occupancy changes in response to JA, focusing on genes whose expression is induced after JA treatment. The ‘JA impact’ column describes how each feature's occupancy is altered following JA exposure. For each epigenomic mark, we also list the known writer and eraser or indicate when their identity remains unknown. −JA and +JA indicate the conditions under which the writer and erasers are active. CRC, chromatin remodeling complex; DNML, DNA demethylase; DNMT, DNA methyltransferase; HDM, histone demethylase; HMT, histone methyltransferase.

A more recent study employing Plant HIgh‐throughput LOw input (PHILO) ChIP‐seq uncovered a highly dynamic H3K9ac landscape: JA‐induced genes displayed increased H3K9ac occupancy (Fig. 1), whereas JA‐repressed genes showed decreased occupancy over time (Choudhary *et al*., 2024). This study also discovered a JA‐induced increase of H3Kac in regulatory regions of JA genes surrounding MYC2 binding sites in both *Arabidopsis* and tomato (Fig. 1), which were termed Stimulus‐Induced Enhancer Acetylation (SIENA) regions (Choudhary *et al*., 2024). Noteworthy is also the JA‐responsiveness of the 3D epigenome highlighted by JA‐induced chromatin looping between enhancer elements and gene bodies of key JA genes (Fig. 1; Wang *et al*., 2019).

Methylation of histone H3 at distinct lysine residues, specifically H3K4me3, H3K36me3, and H3K27me3, is another critical epigenome feature in plants (Cheng *et al*., 2020; Table 1). H3K4me3 and H3K36me3 are associated with actively transcribed genes and H3K27me3 is essential to Polycomb Repressive Complex 2 (PRC2)‐mediated gene silencing (Cheng *et al*., 2020). During active JA signaling, H3K4me3 ChIP‐seq analyses revealed increased H3K4me3 occupancy around the +1 nucleosome of JA‐induced genes (Fig. 1; Zander *et al*., 2020). In addition, JA treatment and infections with necrotrophic fungi lead to an increase in H3K36me3 levels at JA genes in *Arabidopsis* and tomato (Berr *et al*., 2010; Bvindi *et al*., 2022). Also, during *de novo* root regeneration (DNRR), jasmonates act as wound signals that induce H3K36 trimethylation at JA genes (Zhang *et al*., 2019).

The repressive histone mark H3K27me3 appears to play only a minor role in JA signaling, as only *c*. 5% of JA‐repressed genes exhibit JA‐induced H3K27me3 enrichment (Vincent *et al*., 2022). By contrast, JA‐induced removal of H3K27me3 at JA genes has been reported during anther and pollen development in *Arabidopsis* and cotton (Li *et al*., 2021, 2023). Moreover, the repressive histone variants H2A.Z also play crucial roles in the environmental responsiveness of epigenomes (Zander *et al*., 2019; Willige *et al*., 2021). H2A.Z is incorporated into chromatin by the SWR1 complex (SWR1c), and mutations in all three *H2A.Z* genes or SWR1c subunits result in upregulation of JA genes (March‐Diaz *et al*., 2008; Coleman‐Derr & Zilberman, 2012; Berriri *et al*., 2016; Table 1). JA induces extensive reprogramming of the H2A.Z landscape (Fig. 1), characterized by reduced H2A.Z occupancy at JA‐induced genes and increased H2A.Z occupancy in JA‐repressed genes (Zander *et al*., 2020). lncRNAs have emerged as important epigenome regulators, with nearly 10% of expressed lncRNAs being JA‐responsive in tobacco (Zheng *et al*., 2022). Overexpression of the lncRNA *AN LEAF EXPRESSED AND XOO‐INDUCED lncRNA 1* confers resistance to bacterial leaf blight in rice by activating the JA signaling pathway (Yu *et al*., 2020)

The role of DNA methylation in establishing the JA‐responsive core epigenome is poorly understood. JA‐induced immune memory requires components of the DNA (de)methylation machinery (RNA Polymerase V and REPRESSOR OF SILENCING1 (ROS1)); however, differentially methylated regions (DMRs) are confined to specific transposable elements (TEs) rather than JA defense genes (Wilkinson *et al*., 2023). Interestingly, in rice, inhibition of DNA methylation at miniature inverted‐repeat TEs near *JAZ* genes led to their upregulation (Du *et al*., 2025). In addition, overexpression of *DOMAINS REARRANGED METHYLTRANSFERASE 2* (*DRM2*) results in increased CHH (DNA methylation context where H can be adenine, thymine, or cytosine) DMRs at the JA biosynthetic gene *ALLENE OXIDE SYNTHASE*, leading to its transcriptional upregulation (Zhang *et al*., 2025).

## III. Regulators of epigenome responsiveness

The remarkable plasticity of plant epigenomes exhibited upon JA‐Ile accumulation in the nucleus largely depends on operational MYCs. Loss of MYC2 compromises H3K4me3 deposition and results in a markedly altered 3D chromatin conformation including the loss of JA‐induced chromatin looping at several JA genes (Wang *et al*., 2019; Zander *et al*., 2020; Deng *et al*., 2023). While the dynamic establishment of H3K9ac is only moderately affected in *myc2* single mutants, it is strongly impaired in *myc234* triple mutants (Choudhary *et al*., 2024). Collectively, this demonstrates that DNA binding by MYCs constitutes the critical initiating step in JA‐induced epigenome reprogramming (Fig. 1).

**Fig. 2:**
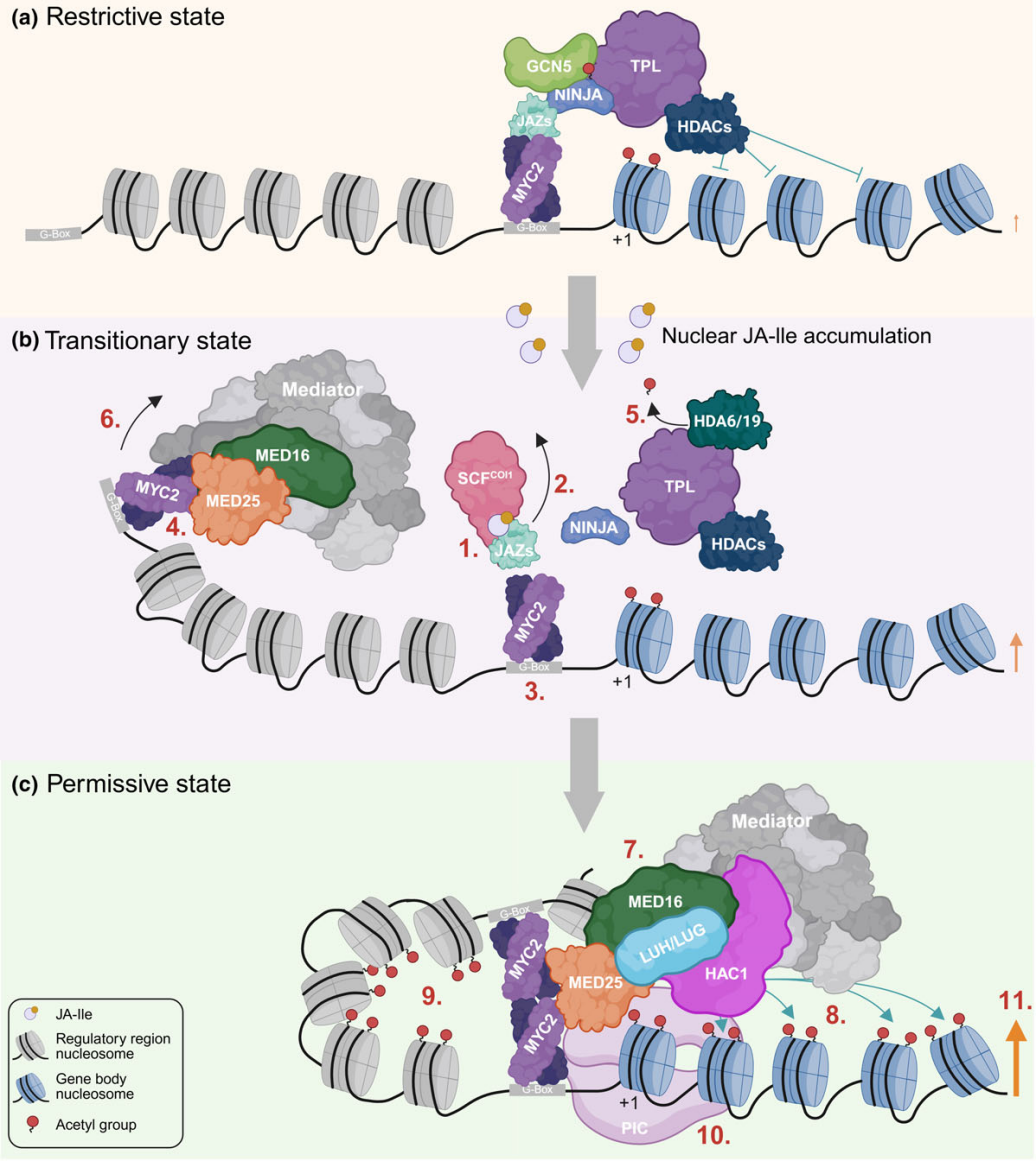
Jasmonic acid (JA)‐induced reprogramming of the histone acetylation landscape. The illustration depicts the chromatin state transition at a representative JA‐inducible gene – from a restrictive to a permissive configuration. Three sequential states are shown: (a) the restrictive state, (b) the transitionary state triggered by nuclear perception of (+)‐7‐iso‐jasmonoyl‐L‐isoleucine (JA‐Ile), and (c) the permissive state associated with robust JA‐induced transcription. In the restrictive state, MYCs are bound to the restrictive TOPLESS (TPL) complex, in which JAZ proteins link MYCs to TPL via NOVEL INTERACTOR OF JAZ (NINJA). GENERAL CONTROL NONDEREPRESSIBLE 5 (GCN5)‐mediated acetylation of TPL, which stabilizes NINJA‐TPL interactions, is indicated. The involvement of currently unidentified TPL‐associated histone deacetylases (HDACs) that deacetylate gene bodies of JA‐responsive genes to dampen active JA signaling is also shown. HDAC‐mediated histone deacetylation is indicated as green blunt‐ended arrows. The transition state highlights the key reprogramming events that occur upon JA‐Ile accumulation in the nucleus, numbered in sequence for clarity: JA‐Ile is perceived by the SCF^COI1^‐JAZ co‐receptor complex (1) and JAZ repressors are ubiquitinated and targeted for proteasomal degradation (indicated by black arrow) (2). Degradation of JAZs releases MYCs from JAZ repression and the TPL complex (3). MYELOCYTOMATOSIS 2 (MYC2) rapidly induces its own transcription, increasing MYC2 dosage and enhancing DNA binding at additional CREs (4). JA‐induced HISTONE DEACETYLASE 6 (HDA6)/19 expression promotes TPL deacetylation, weakening TPL‐NINJA interactions (indicated by black arrow) (5). Liberated MYC2 associates with the MED25–MED16–Mediator complex, facilitating chromatin looping (indicated by black arrow) that brings distal enhancer elements into proximity with JA gene promoters (6). As part of the fully established looped three‐dimensional chromatin structure (7), robust histone acetylation occurs both within the gene body (indicated by green arrows) (8) and at regulatory regions (Stimulus‐Induced Enhancer Acetylation (SIENAs)) (9). This highly permissive chromatin environment facilitates preinitiation complex (PIC) assembly (10) and enables strong transcriptional activation (11). The key steps are highlighted in numerical order for clarity; however, it should be noted that many of these processes occur in parallel, and numerous molecular components remain to be identified. Size of orange arrows indicates level of gene expression, and +1 nucleosome is indicated with +1. This figure was created in BioRender (https://BioRender.com/ndm5rpi).

TFs function as recruitment platforms for chromatin regulators (CRs). For histone acetylation dynamics, these CRs typically include the epigenome writers – histone acetyltransferases (HATs) and the epigenome erasers – HDACs, which exist within multiprotein complexes capable of (de)acetylating multiple lysine residues (Chen *et al*., 2024; Table 1). Current models propose that in the absence of JA, MYCs associate via JAZ‐NINJA or potentially via JAZ‐ECAP (EAR motif‐Containing Adaptor Protein) with the repressive TPL complex comprised of TPL, TOPLESS‐RELATED (TPR) proteins and HDACs to keep acetylation levels at MYC2 target genes low (Fig. 2; Krogan *et al*., 2012, Li *et al*., 2020). However, the HDAC(s) responsible for deacetylating H3ac or H4ac at MYC2 target genes are still unknown. Recent work revealed that TPL's corepressor activity is modulated by direct acetylation through the HAT GENERAL CONTROL NONDEREPRESSIBLE 5 (GCN5) and by JA‐induced HDA6‐mediated deacetylation explaining the counterintuitive JA phenotypes observed in *hda6/19* and *gcn5* mutants (Fig. 2; An *et al*., 2022). Simultaneous loss of *TPL1*, *TPR1*, and *TPR4*, or of 10 *JAZ* genes, leads to a more rapid H3K9ac accumulation after 1 h of JA exposure, confirming their roles as negative regulators of acetylation (Choudhary *et al*., 2024). Intriguingly, H3K9ac levels return to baseline after JA withdrawal in these mutants, implying the action of additional, unidentified HDACs (Choudhary *et al*., 2024).

The permissive MED25 complex that drives H3 acetylation comprises MED25, a subunit of the Mediator complex, together with HISTONE ACETYLTRANSFERASE OF THE CBP FAMILY1 (HAC1) and the Gro/Tup1 family proteins LEUNIG (LUG) and LEUNIG_HOMOLOG (LUHAn *et al*., 2017, Chen *et al*., 2012, You *et al*., 2019, Wang *et al*., 2019). In addition, MED25 is stabilized through its interaction with MED16, which recruits MED25 to the Mediator complex (Fig. 2; Wu *et al*., 2025). Intriguingly, perception of JA‐Ile by the SCF^COI1^‐JAZ co‐receptor complex occurs directly on chromatin, which allows MYC2 to physically associate with the MED25‐HAC1‐LUH complex (Fig. 2; An *et al*., 2017, Chen *et al*., 2012, You *et al*., 2019). This complex is also essential for JA‐promoted petal abs*cis*sion (Furuta *et al*., 2024). Loss‐of‐function mutations in *MED25* and *HAC1* only attenuate JA‐responsive gene expression and moderately reduce H3K9 acetylation, indicating that additional HATs and cofactors contribute to full transcriptional activation (An *et al*., 2017; You *et al*., 2019; Choudhary *et al*., 2024). In sum, this nuclear JA‐Ile‐dose‐dependent switch between MYC‐interacting TPL and MED25 complexes elegantly illustrates how an environmental cue reprograms plant epigenomes (Fig. 2).

Mechanistic insights into JA‐regulated H3K4me3 and H3K36me3 modifications remain limited. Findings in *Arabidopsis* suggest that H3K36 trimethylation is mediated by the histone methyltransferase *SET DOMAIN GROUP 8* (SDG8), as *sdg8* mutants exhibit reduced H3K36me3 levels following *Alternaria brassicicola* infection and during DNRR wound signaling at several marker genes (Berr *et al*., 2010; Zhang *et al*., 2019; Table 1). In tomato, double mutants deficient in *SDG33* and *SDG34* also display decreased H3K36me3 and H3K4me3 levels after *Botrytis cinerea* infection (Bvindi *et al*., 2022). The H3K4me3 reader ALFIN‐LIKE 6 (AL6), a plant homeodomain protein, is also involved in active JA signaling in etiolated seedlings; however, its precise function remains unclear (Vélez‐Bermúdez & Schmidt, 2021). JA‐induced removal of H3K27me3 is a consequence of JAZ‐mediated transcriptional repression via the direct interaction of JAZs and NINJA with PRC2 core components and the H3K27me3 reader *LIKE HETEROCHROMATIN PROTEIN 1 (*LHP1) (Li *et al*., 2021; Table 1). Upon JA signaling, JAZ degradation leads to a loss in PRC2 recruitment, reduction in H3K27me3, and subsequent transcriptional activation of target genes (Li *et al*., 2021). LHP1‐mediated gene repression via H3K27me3 has also been demonstrated for MYC2‐regulated immune TFs (Ramirez‐Prado *et al*., 2019).

The mechanism of JA‐induced chromatin looping remains incompletely understood, but it is increasingly clear that *MYCs* play a central role. MYC2 can form stable *cis* homotetramers *in vitro*, enabling simultaneous binding to two adjacent G‐box elements through DNA looping (Lian *et al*., 2017). In *myc2 Arabidopsis* mutants, JA‐induced chromatin looping at several JA genes is disrupted, and genome‐wide H3K4me3 Chromatin Interaction Analysis with Paired‐End Tag sequencing (ChIA‐PET) analyses have shown extensive alterations of the nucleome (Wang *et al*., 2019; Deng *et al*., 2023). Moreover, recent studies demonstrated that communication between the distal enhancer *GAME Enhancer 1 (GE1*) and the *GLYCOALKALOID METABOLISM (GAME*) gene cluster is mediated by the *MYC2‐GAME9* transcriptional complex, most likely through chromatin looping (Bai *et al*., 2024). Since plant genomes lack CTCF homologs, 3D chromatin organization must rely on alternative mechanisms, one of which involves TF‐Mediator complexes (Fig. 2). Consistent with this, *MYC2* interacts with MED25, and chromatin looping is partially impaired in *med25* mutants (Wang *et al*., 2019).

## IV. Conclusions

Although JA signaling cannot serve as an exact blueprint for other signaling pathways and many open questions remain, four general principles can be broadly applied. First, epigenome reprogramming is extensive for most features, and every gene whose expression changes in response to environmental cues will undergo changes in its local chromatin environment (Fig. 1). However, which of these changes are causal or consequential of cue‐induced transcription is not fully understood. Changes in TF binding, chromatin accessibility, chromatin looping, and H2A.Z occupancy precede cue‐induced transcription and are likely causal (Fig. 1). However, cue‐induced changes in gene body‐localized histone marks such as H3K4me3, H3ac, and H3K36me3 are to some extent the consequence of transcription. Thus, due to the functional coupling, it is not surprising to observe a strong correlation between gene expression and high levels of active histone marks.

Second, environmental responsiveness of the epigenome is mediated by cue‐activated TFs, which is underscored by the lack of stimulus‐induced epigenome reprogramming in TF‐deficient mutants. Beyond *myc* mutants, higher‐order *pif* mutants fail to exhibit low R:FR light‐induced H2A.Z eviction and H3K9 acetylation at *PIF7* target loci (Willige *et al*., 2021). Likewise, *ein3 eil1* double mutants are unable to initiate ethylene‐induced histone acetylation (Zhang *et al*., 2016). Also, cold‐triggered H3K27me3 deposition at the nucleation region of the floral repressor *FLC* is impaired in seedlings lacking the two TFs VIVIPAROUS1/ABI3‐LIKE1 (VAL1) and VAL2, further emphasizing the essential role of TFs in initiating cue‐induced epigenome reprogramming (Yuan *et al*., 2016).

Third, TFs serve as molecular platforms that recruit CRs into regulatory complexes, which directly interface with the RNAPII transcription machinery to integrate upstream cues into gene expression (Smaczniak *et al*., 2012). Because most CRs lack intrinsic DNA‐binding specificity, TFs provide sequence recognition that anchors these complexes to precise genomic loci, enabling spatiotemporal control of epigenome reprogramming (Fig. 2). Each TF associates with distinct CR subsets; notably, PHYTOCHROME‐INTERACTING FACTORs (PIFs) interact with over 10 CRs, illustrating the extensive combinatorial flexibility of TF‐CR modules in regulating the epigenome (Ammari *et al*., 2024).

Fourth, remodeling of the 3D chromatin architecture constitutes an integral feature of the response to environmental cues. Although still relatively underexplored, accumulating evidence suggests that chromatin looping is often a prerequisite for cue‐induced transcriptional regulation. Even within the compact *Arabidopsis* genome, where regulatory regions are relatively short, TF binding sites often reside several kilobases away from their cognate genes, as exemplified by PIF7 binding at the *ARABIDOPSIS THALIANA HOMEOBOX PROTEIN 2 (ATHB2)* locus, underscoring the importance of long‐range enhancer‐promoter communication (Willige *et al*., 2021). Notably, chromatin looping at the large regulatory region of *ATHB2* has been experimentally demonstrated (Kim *et al*., 2021). Similarly, in tomato, heat stress triggers extensive reorganization of the 3D epigenome, partially mediated by the TF HEAT SHOCK FACTOR A1a (HSFA1) (Huang *et al*., 2023).

## V. Perspectives

Despite these advances, key questions remain unresolved: how is the coordinated activity of CRs orchestrated to reprogram multiple epigenome features in parallel? Which additional factors contribute to the establishment, maintenance, and attenuation of epigenome responsiveness? And to what extent is this responsiveness evolutionarily conserved across plant lineages? Studies that capture dynamic changes in diverse epigenomic features over time, particularly in relevant mutant backgrounds, are therefore required. Moreover, proteomic approaches aimed at elucidating cue‐specific TF‐CR complex compositions will be essential for advancing our understanding and ultimately enabling the targeted manipulation of epigenome responsiveness.

## Competing interests

None declared.

## Author contributions

MZ conceived the conceptual framework of the manuscript and wrote the manuscript. EV and MZ generated the figures.

## Disclaimer

The New Phytologist Foundation remains neutral with regard to jurisdictional claims in maps and in any institutional affiliations.
